# The Interplay between Oxidative Stress, Exercise, and Pain in Health and Disease: Potential Role of Autonomic Regulation and Epigenetic Mechanisms

**DOI:** 10.3390/antiox9111166

**Published:** 2020-11-23

**Authors:** Jolien Hendrix, Jo Nijs, Kelly Ickmans, Lode Godderis, Manosij Ghosh, Andrea Polli

**Affiliations:** 1 Pain in Motion Research Group (PAIN), Department of Physiotherapy, Human Physiology and Anatomy, Faculty of Physical Education & Physiotherapy, Vrije Universiteit Brussel, 1090 Brussels, Belgium; Jolien.Hendrix@vub.be (J.H.); jo.nijs@vub.be (J.N.); kelly.ickmans@vub.be (K.I.); 2 Centre for Environment and Health, Department of Public Health and Primary Care, Katholieke Universiteit Leuven, 3000 Leuven, Belgium; Lode.Godderis@kuleuven.be (L.G.); manosij.ghosh@kuleuven.be (M.G.); 3 Department of Physical Medicine and Physiotherapy, University Hospital Brussels, 1090 Brussels, Belgium; 4 Unit of Physiotherapy, Department of Health and Rehabilitation, Institute of Neuroscience and Physiology, Sahlgrenska Academy, University of Gothenburg, 41390 Gothenburg, Sweden; 5 University of Gothenburg Center for Person-Centred Care (GPCC), Sahlgrenska Academy, University of Gothenburg, 41390 Gothenburg, Sweden; 6 Research Foundation—Flanders (FWO), 1050 Brussels, Belgium; 7 External Service for Prevention and Protection at Work (IDEWE), 3001 Heverlee, Belgium

**Keywords:** oxidative stress, exercise, chronic pain, chronic fatigue syndrome, fibromyalgia, autonomic nervous system, epigenetics

## Abstract

Oxidative stress can be induced by various stimuli and altered in certain conditions, including exercise and pain. Although many studies have investigated oxidative stress in relation to either exercise or pain, the literature presents conflicting results. Therefore, this review critically discusses existing literature about this topic, aiming to provide a clear overview of known interactions between oxidative stress, exercise, and pain in healthy people as well as in people with chronic pain, and to highlight possible confounding factors to keep in mind when reflecting on these interactions. In addition, autonomic regulation and epigenetic mechanisms are proposed as potential mechanisms of action underlying the interplay between oxidative stress, exercise, and pain. This review highlights that the relation between oxidative stress, exercise, and pain is poorly understood and not straightforward, as it is dependent on the characteristics of exercise, but also on which population is investigated. To be able to compare studies on this topic, strict guidelines should be developed to limit the effect of several confounding factors. This way, the true interplay between oxidative stress, exercise, and pain, and the underlying mechanisms of action can be revealed and validated via independent studies.

## 1. Introduction

Reactive oxygen species (ROS), including radical (e.g., oxygen, hydroxyl, superoxide ion, nitric oxide) and non-radical (e.g., hydrogen peroxide, peroxynitrite, hypochlorous acid, aldehydes) oxygen species, are pro-oxidant molecules produced during oxygen metabolism. Although their production is a physiological and regulated process [1], high levels of ROS are harmful. In normal circumstances, ROS are cleared by antioxidants. When ROS levels rise due to an increased production and/or decreased clearance, the cell enters a state of stress, called oxidative stress, which potentially leads to lipid, protein, and DNA damage [1,2,3,4].

Oxidative stress is known to be influenced by various stimuli and altered in certain conditions. Two of such stimuli or conditions are exercise and pain. For instance, an imbalance between pro-oxidants and antioxidants has been found in patients with chronic pain, suggesting that it might cover a relevant role in nociceptive processing [5,6,7,8]. Indeed, oxidative stress is capable of inducing deleterious changes, particularly to the central nervous system, given the intrinsic high vulnerability of neurons and glial cells to metabolic changes [9,10], and it has been shown to play an important role in many mechanisms involved in nociceptive modulation and central sensitization [11,12]. Additionally, both a single bout of exercise and physical training affect expression levels of ROS as well as antioxidants [13,14]. Exercises of different type, duration, and frequency have different effects on oxidative stress [14,15], which is in turn related to beneficial or harmful health outcomes. For instance, resistance exercises and aerobic physical activity are beneficial for patients with diabetes mellitus type 1 or 2 [16,17]—which are both conditions where oxidative stress plays a major role (for a review, see [18]). Exercise is also able to influence pain symptoms, and several studies proposed oxidative stress as a mediating factor between exercise and pain [19,20,21,22]; however, findings do not always appear to be consistent. Therefore, we critically review the available literature linking oxidative stress to either exercise or pain separately before diving into the possible interplay between oxidative stress, exercise, and pain. This way, the reader will have a clear overview of evidence on the involvement of oxidative stress and possible confounding factors. As the interplay between oxidative stress, exercise, and pain has never been reviewed before, this narrative review provides a unique compilation of information that might explain concepts such as exercise-induced hypoalgesia and pave the way toward new therapeutic possibilities for chronic pain. In addition, as the underlying mechanisms explaining the interplay between exercise, oxidative stress, and pain are poorly understood, we explain that autonomic nervous system functioning and epigenetic mechanisms hold the potential to unravel the puzzle.

Of important note, that this is a narrative review. We acknowledge that narrative reviews might be prone to selection bias. However, we did base our review on a literature search, which was conducted in Medline using broad search terms (detailed in Appendix A Table A1). To minimize selection bias, the search was primarily focused on systematic reviews, meta-analyses, and recent articles not included in the systematic reviews. Publications on humans were preferred over animal studies. However, animal studies were also selected in case human studies on a specific matter were not available, aiming to review all possible interactions between oxidative stress, exercise, and pain that are currently available.

## 2. The Effect of Exercise on Oxidative Stress in Healthy People

Since the first observation on the topic appeared—42 years ago [23]—our knowledge on the relation between oxidative stress and exercise has significantly expanded. However, it is not yet clear whether exercise-induced oxidative stress is beneficial or harmful to health [24]. The relation between exercise and oxidative stress is not always straightforward but related to many aspects [25]. First, sampling time is crucial for oxidative stress measurement, as biomarkers indicative of oxidative stress or antioxidant systems seem to respond to exercise in a time-specific manner [26]. For instance, thiobarbituric acid reactive substances (TBARS) and protein carbonyl levels, two commonly used biomarkers indicative of oxidative stress, reach a peak at one and four hours after a single bout of exercise, respectively [26]. Secondly, oxidative stress is the result of complex interactions, and many different biomarkers have been used to assess it. When reading the research on oxidative stress, it is important to consider that different biomarkers aimed at measuring the same oxidative status might lead to different results, depending on their specific function [27]. Illustratively, lymphocyte glutathione peroxidase and catalase activities (markers for antioxidant capacity) increased in response to moderate exercise while superoxide dismutase (SOD, a marker for antioxidant capacity) activity remained stable [27]. In addition, the characteristics of exercise as well as a person’s nutritional status and physical activity level may have an effect and lead to different results [15,27,28,29,30]. In the next sections, we review oxidative stress and antioxidant capacity changes in response to a single session of intense or moderate exercise, and to more prolonged physical training. The results of these sections are summarized in Table 1.

### 2.1. A Single Bout of Intense Exercise and Oxidative Stress

Exercise intensity is commonly defined by using a percentage of the maximum oxygen uptake (%VO_2max_) [33]. However, precise cut-offs to classify exercise intensities are lacking, as the existing literature provides multiple ranges for measures indicating exercise intensity [34,35]. As a result, studies investigating the same exercise intensity in terms of definition (i.e., low, moderate, or high) might not use the same %VO_2max_. Therefore, this review reports the exact exercise intensity in between brackets.

The effect of a single bout of intense exercise on oxidative stress status has been explored in several conditions such as cycling, intermittent running, sprints, jumps, and swimming in both animal models and humans [28,36,37,38,39]. An intense swimming session (75—80% of individual maximal capacity) of one hour increased malondialdehyde (MDA, a marker for lipid peroxidation) and protein carbonyl levels and decreased antioxidant enzyme levels in neutrophils and lymphocytes of amateur swimmers [36]. Another study implementing exercise until exhaustion also found indicators of increased oxidative stress, which was expressed via increased TBARS levels and decreased reduced glutathione (GSH, inversely related to oxidative stress) [38].

Of note, when comparing intense and moderate exercise, Wang and Huang found intense exercise (80% of VO_2max_) to cause a reduction in GSH, while this was not the case for moderate exercise (60% of VO_2max_), indicating that oxidative stress changes in response to exercise might be dose-dependent [15]. On the contrary, a more recent study concluded that all exercise intensities (low (40% of VO_2max_), moderate (60% of VO_2max_), and high (80% of VO_2max_)) significantly increased MDA levels immediately after exercise without being significantly different from each other [40]. However, these studies did not assess the same markers for oxidative stress status and thus, they are difficult to compare, as different markers for oxidative stress status respond differentially to exercise [26,27].

In general, the literature suggests that high intensity, strenuous exercises can increase oxidative stress in both trained and untrained people [28,36,38]. Furthermore, parameters reflecting antioxidant status (e.g., glutathione and total antioxidant capacity) decrease immediately after exhaustive exercise [26,38,41,42] and rapidly increase in the recovery phase, starting at approximately 30 min after activity [41,43].

### 2.2. A Single Bout of Moderate Exercise and Oxidative Stress

Existing literature linking oxidative stress changes to moderate exercise is less consistent. While some reported a marked increase in blood markers for oxidative stress after submaximal cycling [44,45], swimming [46], or resistance training [47,48], others did not [15,49,50,51,52]. Moreover, several studies found that submaximal exercise increased total antioxidant status in active [45,48,52] as well as sedentary individuals [45,53,54]. However, the results of a recent study are not in line with this, as they show that moderate exercise (60% of VO_2max_) did not increase SOD levels, whereas intense exercise (85% of VO_2max_) did [55].

Most part of these conflicting results is likely to be explained by differences in participants’ training level (see the next section for more details) [49] or physical condition [56,57], or by insufficient exercise intensity [15,50,58]. For instance, moderate exercise (70% VO_2max_) increased glutathione reductase levels in young, sedentary, severely obese volunteers, whereas no significant change was observed in overweight/moderately obese or normal-weight individuals [56]. Additionally, moderate exercise (60% of peak workload) increased oxidative stress in patients suffering from cystic fibrosis but not in healthy volunteers [59]. In conclusion, despite inconsistencies in the literature, most research showed that moderate exercise has the potential to induce oxidative stress and increase total antioxidant status [25,53,60,61,62].

### 2.3. Physical Training and Oxidative Stress

Many studies explored the effect that prolonged training might exert on oxidative stress in healthy people. Several studies reported that training alleviates exercise-induced oxidative stress by modulating the antioxidant capacity in humans (for a review, see [63]). However, not all training modalities and intensities have the same beneficial effect. For instance, oxidative stress decreased in rats subjected to high-intensity (85% VO_2max_) interval training, whereas this was not the case for rats subjected to continuous low-intensity training (40% VO_2max_) [14].

The person’s basal training level also influences oxidative stress. Physically active individuals (more than 150 min of exercise per week) are less prone to DNA damage caused by exercise-induced oxidative stress than sedentary individuals [64]. This sort of decreased vulnerability to oxidative damage is probably the result of coping mechanisms taking place in response to oxidative stress during the recovery phase, including the activation of antioxidant and oxidative damage repair systems mediated via the increased ROS production. As a detailed description of how pro-oxidants induce such adaptations is beyond the scope of this review, we refer interested readers to a review by Gomez-Gabrera [65]. These endogenous coping mechanisms increase the tissue’s ability to cope with oxidative stress/damage [66,67]. Hence, physical training has the potential to induce oxidative stress and underlying chemical alterations that are linked to beneficial health outcomes (i.e., oxidative eustress) (for a review, see [68]). This seems particularly true for moderate exercise training [69]. Conflicting results were found when investigating aerobic exhausting exercise, anaerobic exercise, or a combination of the two [69]. Nevertheless, more recent studies found that 4 to 12 weeks of high-intensity training (80% of maximal heart rate (%HR_max_)) reduced oxidative stress at baseline and following exhausting exercise in untrained people [70] and athletes [71].

In practice, the adaptations occurring in response to training result in higher levels of several antioxidant enzymes in physically active people at rest [45,72,73]. Furthermore, while both trained and untrained people showed an increase of MDA levels after exercise (cycling until exhaustion), only the trained ones demonstrated a significant increase of SOD, vitamin E, and glutathione peroxidase in the recovery phase [73]. However, too intense training can compromise the antioxidant response, even in athletes. Four weeks of overload training induced an increase of TBARS levels and a decrease in total antioxidant status, in both rest and exercise conditions [74]. At this stage, physical training results in oxidative distress instead of oxidative eustress, leading to the loss of beneficial health outcomes related to physical training. Thus, depending on the characteristics of exercise, physical training will induce oxidative eustress or distress, leading to beneficial or harmful chemical adaptations and eventually health outcomes, respectively (for a review, see [68]).

Of important note, oxidative stress has classically been seen as a dangerous phenomenon [24]. Antioxidant supplementation is commonly used among athletes and physically active people in the attempt of reducing exercise-induced oxidative stress and increasing performance. However, its benefits are unclear [75,76,77,78]. It is possible that antioxidant supplementation mitigates the above-mentioned endogenous coping mechanisms which are activated via oxidative eustress [65,79]. A recent study even demonstrated that moderate training is more beneficial than selenium supplementation (an antioxidant), improving the antioxidant status and decreasing exercise-induced oxidative damage [80]. Therefore, moderate and controlled physical activity should be considered as a valuable strategy to promote desirable changes in the balance between pro- and antioxidant products.

## 3. Oxidative Stress Contributes to Pain

The relation between oxidative stress status and nociception has been evident for decades [81,82,83,84,85]. Following inflammatory stimuli, the production of certain ROS (e.g., H_2_O_2_ (hydrogen peroxides), 2O_2_^−^ (superoxide), and ONOOH (peroxynitrite)) is increased to mediate various aspects of the immune response [86,87]. Moreover, oxidative mechanisms mediate thermal and mechanical hyperalgesia induced by nerve growth factor injections in both peripheral and central nerve fibers. Nerve growth factor increases the production of oxidized lipids, which in turn modulate transient receptor potential vanilloid 1 (TRPV1) activity [88,89]—a multimodal receptor known for its involvement in the transduction of nociceptive, thermal, and acidic stimuli [90]. Additionally, elevated spinal levels of ROS can alter nociception and lead to the hyperexcitability of both the peripheral and central nervous system (i.e., referred to as peripheral and central sensitization, respectively), resulting in hyperalgesia without any nerve damage or tissue inflammation [91,92]. Finally, nitric oxide reduces receptor thresholds, resulting in peripheral and central sensitization [93], and the inhibitory activity of the central nervous system, leading to central sensitization of the dorsal horn neurons [94]. In line with the above observations, oxidative stress has also been associated to chronic pain and proposed to be a possible contributor to the maintenance of pain symptoms [20,84,95,96]. While both inflammatory and oxidative processes cover a role in the acute nociceptive phase, it is mainly the production of ROS that seems to account for the maintenance of nociceptive processes in the chronic phase [97]. These observations linking oxidative stress status and nociception stimulated researchers to investigate the potential analgesic properties of oxidative stress-modulating compounds and oxidative stress status in various pain populations, as reviewed in the next sections.

### 3.1. Oxidative Stress in Chronic Pain Populations

Several underlying mechanisms will likely contribute to the pathophysiology of complex pain syndromes such as tension-type headache and complex regional pain syndromes [98,99,100,101]. Available research demonstrated that higher MDA levels are present in the serum and saliva of people suffering from chronic regional pain syndrome [5]. Similarly, patients with tension-type headache showed higher plasma levels of MDA and TBARS compared to healthy pain-free individuals [6]. Taken together, these findings suggest an involvement of oxidative stress mechanisms in the pathophysiology of such conditions. Myalgic encephalomyelitis/chronic fatigue syndrome (ME/CFS) and fibromyalgia (FM)—two conditions in which widespread persistent pain is a defining symptom—have been extensively investigated with regard to oxidative stress. Research showed evidence of increased oxidative stress in both patients and animal models (for a review, see [7,8]). Complex conditions such as ME/CFS and FM are very challenging to be translated in animal models given the poor understanding of their etiology (for a review, see [102,103]). However, animal models mimicking the most important features of FM, including depressive- and anxiety-like symptoms, have recently been proposed [104,105,106]. In the reserpine-induced FM model, parameters indicative of oxidative stress were found to be altered in cerebrospinal fluid (CSF) [107], brain tissue [108], spinal cord, and muscles [105].

Results of clinical research are in line with those obtained from animal studies. Sanchez-Dominguez and colleagues collected skin biopsies from patients with fibromyalgia and healthy controls, finding significant mitochondrial dysfunction and increased levels of oxidative stress [109]. Indications of oxidative stress were also found in other tissues and fluids, such as plasma, serum, erythrocytes, and mononuclear cells in patients suffering from ME/CFS [7,110,111,112,113,114,115]. These patients showed higher levels of oxidized low-density lipoproteins and protein carbonyl, which are both a result of oxidative stress [114]. Blood parameters indicative of oxidative stress were also associated with symptoms, including pain [81]. This has also been observed in animal models [116]. In line with these observations, several studies reported reduced Coenzyme Q10 (CoQ10) levels [109,117,118,119], which is vital for mitochondrial functioning [120] and thus may be a potential cause of impaired mitochondrial functioning and increased oxidative damage [121].

### 3.2. Decrease of Pain via Down-Regulating Oxidative Stress

Numerous studies explored the effect of oxidative stress-modulating compounds on pain and found that pain decreases when oxidative stress is down-regulated [122]. For instance, the effect of CoQ10 supplementation was assessed in several clinical trials, which found that CoQ10 supplementation restored biochemical parameters and induced a significant improvement in pain, fatigue, headache, psychopathological, and depressive symptoms in patients with FM [123,124,125,126,127,128]. Recently, these observations were confirmed in vivo via the use of the reserpine-induced FM model [105]. CoQ10 supplementation also yields favorable results in other pain populations (e.g., statin-associated myalgia and migraine) [129,130] and models (e.g., neuropathic pain and osteoarthritis) [131,132,133]. Other antioxidants have also been investigated. For instance, SOD mimetics down-regulate oxidative stress via its ability to neutralize superoxide and therefore exert an antinociceptive effect in inflammatory as well as drug-induced pain models [85,91,134]. Next to the analgesic effect of antioxidants, other compounds are also implicated to alleviate pain via its effect on oxidative stress. Inhibitors of electron transport chain complexes, which are major ROS producers, decreased pain in neuropathic and inflammatory pain models [135]. Additionally, it has recently been demonstrated that activators of Nuclear factor-erythroid 2-related factor 2 (Nrf2), a master regulator of antioxidant defense [136], significantly reduce pain and delay the onset of pain in various pain models [136,137,138,139,140,141,142].

In conclusion, this body of evidence, including oxidative stress status in pain populations as well as the favorable effect of down-regulating oxidative stress in these populations, further supports the fact that oxidative stress is involved in nociceptive modulation.

## 4. Interactions between Oxidative Stress, Exercise, and Pain

**Figure 1:**
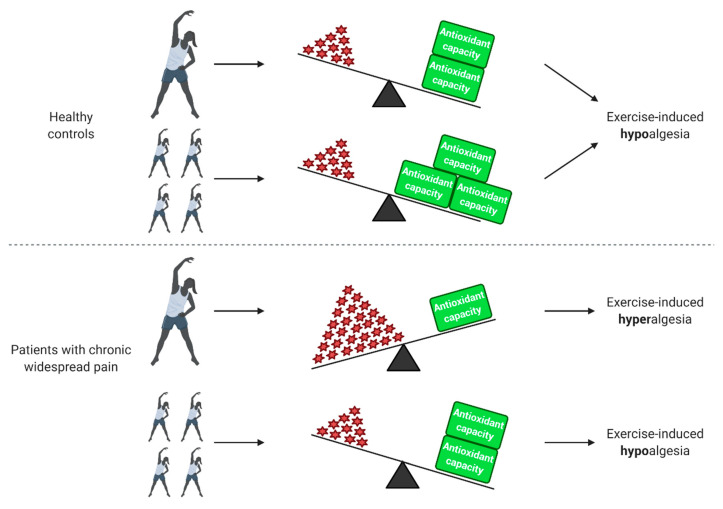
Effect of exercise on healthy people and patients with chronic widespread pain in relation to oxidative stress status and pain. In healthy individuals, a single bout of exercise increases reactive oxygen species (ROS) production, which in turn results in an increased antioxidant capacity during the recovery period. After physical training, the antioxidant capacity of healthy people increases. Both a single bout of exercise and physical training have a hypoalgesic effect in healthy individuals. Patients with chronic widespread pain already have elevated ROS levels at baseline and an impaired antioxidant capacity. In these patients, a further increase in ROS induced by a single bout of exercise is linked to exercise-induced hyperalgesia. Physical training decreases baseline ROS levels and improves antioxidant capacity, leading to a decrease of oxidative stress and hypoalgesic effect. Created with BioRender.com.

Although several studies investigated the relation between exercise and chronic pain [143,144,145,146,147,148,149], only a small number also inquired information about the role of oxidative stress. Figure 1 summarizes the effect of exercise on healthy controls and patients with chronic widespread pain in relation to oxidative stress status and pain.

### 4.1. Exercise-Induced Hypoalgesia in Healthy Controls and Various Pain Populations

Several studies reported that exercise decreases pain threshold and intensity, a phenomenon known as exercise-induced hypoalgesia, in healthy controls as well as various pain populations, including chronic low back pain, rheumatoid arthritis, and osteoarthritis (for review see [150]). The phenomenon of exercise-induced hypoalgesia has been linked to oxidative stress status. Illustratively, Chen and colleagues reported that an exercise protocol (muscle stretch and strengthening exercises) significantly decreased pain in patients with nonspecific low back pain, which was accompanied with decreased hydrogen peroxide and increased SOD and catalase activity [19]. Animal studies confirm this link between exercise-induced hypoalgesia and oxidative stress. In a model of neuropathic pain, mechanical allodynia (i.e., painful response to non-painful stimuli) and thermal hyperalgesia (i.e., increased pain response to painful stimuli) were prevented in rats that followed physical training (70% VO_2max_) after surgery but not in rats that followed the same training before surgery. Interestingly, total antioxidant capacity and ferric reducing ability of plasma (FRAP, a marker for antioxidant power) significantly increased in rats that trained after surgery compared to injured rats that did not train. This was not the case for rats that trained before surgery, suggesting that oxidative stress status was involved in exercise-induced hypoalgesia [151].

### 4.2. Exercise-Induced Hyperalgesia in Patients with Chronic Widespread Pain

Although exercise alleviates pain in various pain populations via its effect on oxidative stress, patients with chronic and more widespread pain, such as chronic whiplash-associated disorders, FM and ME/CFS, but also some patients with osteoarthritis pain, do not seem to benefit from a single bout of exercise. According to a recent review, most studies comparing pain thresholds before and after exercise in patients with chronic pain as well as healthy controls conclude that exercise-induced hypoalgesia occurs in healthy controls, while exercise-induced hyperalgesia (i.e., increased pain after exercise) is observed in patients with chronic whiplash-associated disorders, FM, and ME/CFS [150]. This may potentially be explained by the fact that these patients have elevated ROS and decreased antioxidant enzyme levels at baseline when compared to pain-free controls. It is possible that a further increase in oxidative stress would contribute to explain the typical exercise-induced symptom exacerbation in these patients [152,153], as confirmed by Jammes and colleagues. They noted that maximal exercise (incremental cycling until exhaustion) induced an increase in TBARS levels and suppressed heat shock proteins—which are known to exert a protective function against ROS—in patients with ME/CFS that was more pronounced and appeared earlier than in healthy sedentary controls [20,21,22]. Moreover, they reported that oxidative stress—rather than pro-inflammatory cytokine responses [21,154]—contributed to explain muscle pain and post-exertional malaise [22].

In contrast to what happens during a single bout of exercise and in line with the evidence in healthy people, regular physical activity exerts a desirable effect on oxidative stress and health in these patients. Twelve weeks of regular exercise (65–85% of %HR_max_) seems beneficial in patients with fibromyalgia while simultaneously reducing oxidative stress markers (TBARS, protein carbonyls, nitric oxide, MDA), increasing antioxidant capacity (catalase, thiol, glutathione peroxidase, β-carotene, vitamin A, and vitamin E) and improving symptoms [155,156,157]. Accumulating evidence showed that regular moderate aerobic exercise is beneficial for patients with chronic widespread pain [152].

## 5. The Potential Role of the Autonomic Nervous System in the Interplay between Oxidative Stress, Exercise and Pain

**Figure 2:**
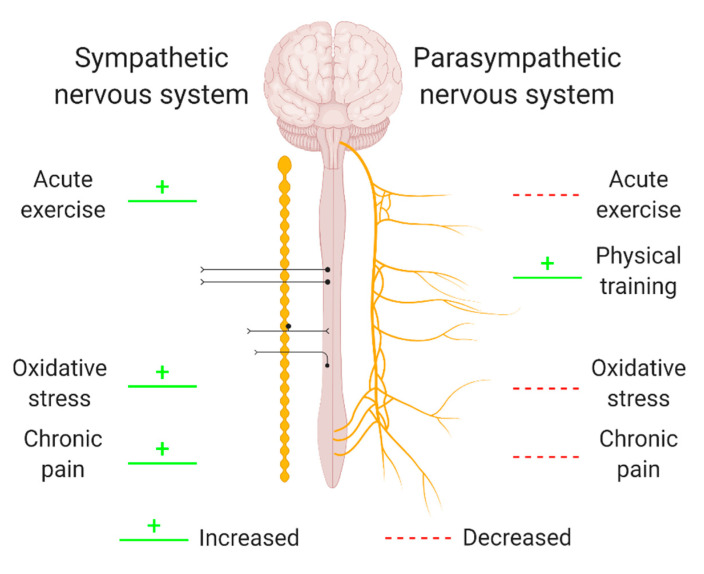
The relation between oxidative stress, exercise or pain and different branches of the autonomic nervous system. Oxidative stress, exercise, and pain have been related to an altered activity of the sympathetic branch (left) and the parasympathetic branch (right) of the autonomic nervous system (ANS). As the directionalities of these relations are not yet clear, green and red lines indicate a non-directional relation between ANS activity and exercise, oxidative stress, or chronic pain. Hence, changes in ANS activity might be a regulator of and/or a response to exercise-induced adaptions, oxidative stress, and chronic pain. Created with BioRender.com.

The autonomic nervous system (ANS) is a control system regulating a variety of crucial body functions such as cardiac, respiratory, and vasomotor functions. Its branches supply internal organs, muscles, and the skin and influence their function by releasing neurotransmitters such as acetylcholine, adrenaline, and noradrenaline [158]. Given its broad extension and numerous extensions, the ANS is able to influence stress responses, the immune system, and inflammation [159]. In addition, ANS activity changes have been related to oxidative stress, exercise, and chronic pain (Figure 2) [160,161,162,163,164,165,166,167,168]. However, whether ANS activity regulates and/or responds to exercise-induced adaptions, oxidative stress, and chronic pain is yet to be properly investigated, as only one study was found to investigate these links simultaneously.

As previously mentioned, a number of adaptions occur in response to exercise, which result in changes in ANS activity. More than 60 years ago, Van Liere et al. found that propulsive motility of the small intestine was higher in exercised rats compared to sedentary rats, which was possibly due to an increased parasympathetic tone, and therefore, they were one of the first groups reporting ANS activity changes in response to exercise [169]. Since then, many others investigated this relation and reported that acute exercise tends to reduce cardiac vagal modulation (i.e., parasympathetic tone) and increase sympathetic activity, whereas physical training increases the parasympathetic tone (for a review, see [168]). Elevated sympathetic tone has the potential to impair local microcirculation and possibly cause painful ischemia [170,171,172]. As a result, sympathetically-maintained vasoconstriction leads to insufficient blood flow for working muscles, producing muscle hypoxia and increased oxidative stress, which in turn can maintain nociceptive stimuli [173]. On the contrary, increased vagal (parasympathetic) tone is important for post-exercise recovery [174,175,176,177,178,179].

Furthermore, several studies found that changes in ANS activity can also be linked to pain, ranging from an altered ANS activity in patients with chronic pain to correlations between ANS and pain parameters. For instance, increased sympathetic and reduced parasympathetic tone have been reported in patients with chronic pain [160,161,162]. Additionally, blood pressure changes, which is considered a measure for sympathetic activity [180,181,182], have been associated to pain sensitivity [183] and linked to exercise-induced hypoalgesia [184], suggesting a relevant role for the baroreceptor reflex [185]. Moreover, heart rate variability (HRV)—a measure for efferent cardiac vagal nerve activity (i.e., parasympathetic activity) [186]—is inversely correlated with reported pain [187]. In fact, the parasympathetic branch of the ANS exerts anti-inflammatory functions by dampening the release of pro-inflammatory cytokines [188,189,190]. Hence, knowing that pain is one of the cardinal symptoms of inflammation [191], the anti-inflammatory effect of an increased parasympathetic tone could be a potential mechanisms of action underlying the inverse correlation between HRV and reported pain. As a large body of literature demonstrated a close link between inflammation and oxidative stress (for a review, see [192,193,194,195]), parasympathetic branch activity is likely able to regulate both inflammation and oxidative stress simultaneously.

Preliminary evidence suggests that the ANS might act as a mediator of oxidative stress responses [52,174]. Blockage of the angiotensin-II receptor induced both inhibition of the ANS sympathetic branch and reduction of oxidative stress [163]. Additionally, several studies indicate that the relation between the ANS and oxidative stress might be bidirectional, as oxidative stress is also able to modulate ANS activity. Increased oxidative stress in the rostral ventrolateral medulla causes an excitation of the ANS sympathetic branch [163] and experimentally induced increased levels of oxidative stress alter blood flow regulation, thereby reducing muscle blood flow during exercise in both animals and humans [163,164,165,166,167]. As mentioned above, insufficient blood flow for working muscles produces muscle hypoxia and increases oxidative stress, which is in turn linked to pain [173]. Hence, combining findings from independent studies suggests that ANS activity might play a role in the interplay between oxidative stress, exercise, and pain.

In line with this, our group recently investigated the associations between oxidative stress and pain symptoms in relation to exercise in healthy volunteers and people with ME/CFS, and we assessed whether the oxidative stress level and parasympathetic (vagal) activity were linked [196]. Even though exercise did not increase oxidative stress levels, oxidative stress was consistently found to be associated with ME/CFS patients’ pain symptoms both before and after exercise. Furthermore, HRV was strongly associated with oxidative stress reduction in healthy people and remained stable during exercise in healthy volunteers but significantly decreased in patients with ME/CFS [175]. As this association was only found in healthy controls, it might be a normal physiological response that may be disrupted in ME/CFS patients. Although research is not conclusive, the aforementioned studies indicate that ANS activity might cover an important mediating role in the interactions between oxidative stress, exercise, and pain. More research is warranted, as it might hold important clinical implications.

## 6. Genetic and Epigenetic Regulatory Mechanisms

**Figure 3:**
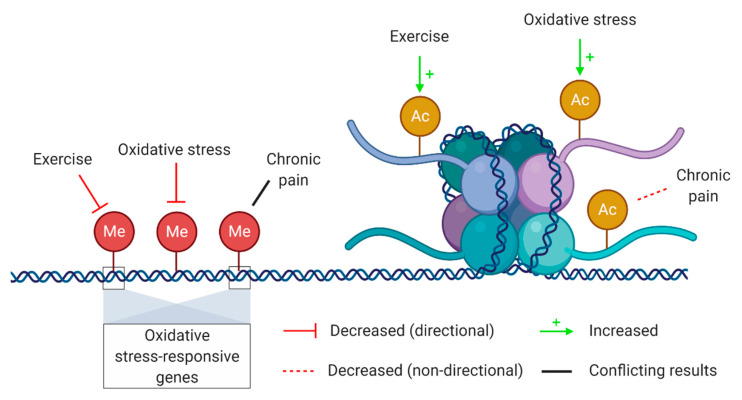
Epigenetic mechanisms potentially involved in the interplay between oxidative stress, exercise, and chronic pain. Epigenetic mechanisms that are potentially involved in the interplay between oxidative stress, exercise and chronic pain include DNA methylation (left, red circles) and histone acetylation (right, yellow circles). A distinction was made between interactions with a proven directionality (solid red and green arrows) and those without (dashed red and solid black line). Thus, exercise and oxidative stress decrease DNA methylation and increase histone acetylation, whereas chronic pain has only been linked to decreased histone acetylation and altered DNA methylation patterns. Created with BioRender.com.

The link between genetics and oxidative stress is bidirectional. On one hand, it has long been known that oxidative stress is able to impair the proliferative capacity of a cell as well as to directly induce DNA damage. On the other hand, genetic changes such as mutations and polymorphisms can influence gene expression and thus those functions regulated by the mutated gene. Thus, any genetic change in mitochondrial DNA influencing mitochondrial functions can potentially have an impact on redox-sensitive sites and their functions [197]. In the last two decades, it has become clear that gene expression and regulation is not solely directed by our genes, but rather by a complex interaction of genetic and epigenetic mechanisms [198]. Epigenetics refers to a set of biological mechanisms able to change gene expression without interfering with the DNA sequence itself [199]. Importantly, epigenetic processes are influenced by environmental and lifestyle factors. Epigenetic adaptations occurring in response to exercise have been extensively investigated as well as epigenetic mechanisms related to oxidative stress, nociception, and pain. However, epigenetic research on the interplay between oxidative stress, exercise, and pain is lacking. An overview of known relations between epigenetic mechanisms and oxidative stress, exercise, and chronic pain is provided in Figure 3.

DNA methylation is one of the most well-known epigenetic mechanisms and may be involved in the interplay between oxidative stress, exercise, and pain. DNA methylation is characterized by the transfer of a methyl group to DNA, mainly to the cytosine base of cytosine–guanine di-nucleotides, via a family of enzymes called DNA methyltransferases (DNMTs) [200]. Methylated DNA is less accessible to transcription factors and thus is associated with reduced gene expression. Research showed that DNA methylation was lower in gene promotors of trained men compared to untrained men. Interestingly, many of these hypomethylated promotors drive the expression of oxidative stress-responsive genes, such as *SOD2* [201]. Several other studies observing altered DNA methylation patterns in physically active people also reported hypomethylation on oxidative stress-responsive genes, leading to an increased expression of those genes and indicating that physically active people cope better with oxidative stress than sedentary individuals [202,203,204,205]. Moreover, DNA methylation patterns showed to be altered in patients with chronic widespread pain compared to healthy people or their unaffected twin (for a review, see [206]). Genes that were differentially methylated were involved in diverse processes such as chromatin packaging, oxidative stress responses, nociceptive and neuropathic signaling, and muscle contraction [207,208].

Another epigenetic mechanism that potentially plays a role in the interaction between oxidative stress, exercise, and pain is histone acetylation. This process is partially mediated via histone deacetylases (HDACs), which is a family of enzymes responsible for removing acetyl groups from histones leading to decreased accessibility of DNA and thus reduced expression. HDACs were found to be exported from the nucleus during exercise [209]. Even more interesting, this export of HDACs was found to be fostered by oxidative stress [210], thereby linking increased oxidative stress in response to acute exercise to exercise-induced epigenetic adaptations. Additionally, increased HDAC levels, and subsequently decreased histone acetylation, have been found in animal models of chronic pain [211,212,213,214]. Interestingly, exercise was able to reverse increased HDAC levels and therefore increase histone acetylation while attenuating mechanical allodynia and thermal hyperalgesia [214]. Hence, increased histone acetylation levels might contribute to explain exercise-induced hypoalgesia.

Not only are expression levels of genes related to oxidative stress influenced by epigenetic mechanisms, oxidative stress itself is also able to induce epigenetic changes, including alterations in DNA methylation and histone acetylation patterns [215,216]. Oxidative stress converts methylated cytosines into hydroxyl-methylated ones, which in turn prevents the maintenance of DNA methylation and promotes gene expression [217,218]. In line with the increased oxidative stress levels observed after a single bout of exercise, this type of exercise induces the hypomethylation of DNA in skeletal muscle of healthy individuals [202]. Oxidative stress is also able to influence chromatin-modulating enzymes, which are frequently redox-sensitive [219]. Expression levels as well as the activity of several chromatin-modulating enzymes (e.g., histone acetyltransferases, HDACs, and DNMTs) are affected by oxidative stress [220,221].

The field of epigenetics holds promise to unravel currently unknown mechanisms of action due to its interplay with several stimuli. Epigenetics could be a common underlying mechanism of action explaining the interactions between oxidative stress response, exercise, and pain. However, research investigating the nature of these interactions is lacking and thus should be conducted in the coming years to uncover the regulatory and/or responsive role of epigenetics processes.

## 7. Future Recommendations

To reveal the true interplay between oxidative stress, exercise, and pain, the scientific community should first agree upon several matters to eliminate many confounding factors limiting advances in this field of research. With regard to oxidative stress measurement, researchers should invest time in determining which biomarkers reflect oxidative stress status most accurately. For instance, even though new and more accurate biomarkers emerged rather recently, those that have been used for decades remain the “golden standard” (for a review, see [222]). Moreover, few studies investigating the link between oxidative stress, exercise, and/or pain targeted more than two markers for oxidative stress, whereas oxidative stress status is determined by the balance among many of them. Therefore, a variety of indexes reflecting oxidative stress status have been proposed [223,224]. These indexes will probably reflect oxidative stress status more accurately and limit false positives or negatives, since they are dependent on several markers indicative of different aspects contributing to oxidative stress status.

As previously mentioned, another important matter to consider when assessing oxidative stress is sampling time. Although Michailidis et al. found that most markers of oxidative stress peak a couple of hours after exercise instead of immediately after, many studies focusing on exercise-induced oxidative stress only assessed oxidative stress markers immediately after exercise [26]. As a result, important information about oxidative stress changes in response to exercise might be neglected, especially when assessing only one or a limited number of markers. Hence, one should always obtain accurate knowledge about the time-specific response to exercise of the oxidative stress markers that will be assessed before deciding upon the optimal sampling time(s).

Additionally, altered plasma concentrations of some antioxidants in response to exercise might reflect a redistribution between tissue and plasma rather than true changes in antioxidant capacity [225]. Thus, markers for oxidative stress and antioxidant capacity should ideally be assessed in plasma as well as different tissue types (e.g., liver and muscle) simultaneously. As the collection of human tissue is an invasive, and in some cases even unethical (e.g., liver), procedure, human studies should be complemented with animal studies to acquire a detailed and complete overview of oxidative stress alterations in response to exercise.

As noted by Dalleck et al., exercise intensity might be the most determining component when aiming to reach beneficial effects of exercise [33]. Therefore, strict guidelines with regard to the definition of different exercise intensities and standardized measures to define them are necessary to allow independent studies to be comparable. Although the American College of Sports Medicine (ACSM) provides such guidelines, including those to reach optimal cardiorespiratory fitness [34], Dalleck et al. found that these guidelines are frequently misused and misinterpreted [33]. For instance, following ACSM’s guidelines, exercise intensity should be defined via the percentage of the heart rate reserve (%HRR) or of the oxygen uptake reserve (%VO_2_R) [34] while %VO_2max_ is most commonly used in the literature [33]. This lack of standardized measures and definitions for exercise intensity is even more important knowing that oxidative stress alterations in response to exercise seem to be dose-dependent [15].

This dose-dependent response of oxidative stress to exercise is also likely to influence exercise-induced and oxidative stress-mediated hypo-/hyperalgesia. Indeed, a rather outdated review indicated that exercise-induced hypoalgesia is dependent on exercise intensity, most likely in combination with exercise duration and modality [226]. Hence, a range of exercise intensities, modalities, and durations should be taken into account when unravelling these phenomena.

Moreover, the occurrence of exercise-induced hypo-/hyperalgesia is dependent on which population is investigated. While exercise seems to induce hypoalgesia in pain-free individuals, results are conflicting when looking at chronic pain populations (for a review, see [150]). Taking into account that oxidative stress levels have been found to be altered in patients with chronic pain [5,6,7,8], the effect of exercise on pain and the mediating role of oxidative stress should be assessed in diverse, but well-defined, pain-free and pain populations.

When the above recommendations are followed, the true interplay between oxidative stress, exercise, and pain will likely become evident. Obviously, these recommendations are time- and money-consuming, but they are also pivotal because there are currently too many variables preventing independent studies to be compared properly. Hence, they will contribute to a better understanding of the complex interactions between exercise, oxidative stress and pain as well as other oxidative stress- or exercise-related questions.

Next to acquiring a clear and detailed overview of the interplay between oxidative stress, exercise, and pain, unravelling the underlying mechanisms of action should also be emphasized as they could provide new therapeutic possibilities for chronic pain. For instance, epigenetic modifications might be able to explain exercise-induced and oxidative stress-mediated hypoalgesia in healthy controls [214,220,221]. Then, eventually, epigenetic editing could possibly be used to induce the same epigenetic modifications in patients with chronic pain, thereby decreasing pain.

## 8. Conclusions

Oxidative stress has been extensively investigated in relation to exercise and pain. However, results of studies from different research groups are challenging to compare, as a range of exercise modalities and intensities have been implemented and many markers have been used to assess oxidative stress and pain. Although the underlying mechanisms are poorly understood, there seems to be a relation between oxidative stress, exercise, and pain. ANS functioning and epigenetic mechanisms appear to mediate these relations, as they have been implicated in oxidative stress, exercise, and pain separately as well as in the interaction between them. Studies focusing on the role of epigenetics in the relation between oxidative stress, exercise, and pain are necessary to confirm their real contribution.

## Figures and Tables

**Table 1:** Overview of different markers to assess oxidative stress, their behavior in response to a single bout of intense exercise, a single bout of moderate exercise and physical training, and their expression in patients with chronic widespread pain.

Category	Commonly Used Markers	Single Bout of Intense Exercise	Single Bout of Moderate Exercise	Physical Training	Chronic Widespread Pain
ROS	H_2_O_2_, NO, O_2_^−^	↑	Conflicting results	↑ ^1^ and ↓ ^2^	↑ ^1,2^
Oxidation products	TBARS, MDA, Protein carbonyls	↑	Conflicting results	↑ ^1^ and ↓ ^2^	↑ ^1,2^
Antioxidant capacity	TAC, SOD, Catalase, GPX, GR	↓	Conflicting results	↑ ^1,2^	↓ ^1,2^

^1^ At rest compared to untrained/pain-free people. ^2^ After a single bout of exercise compared to untrained/pain-free people. ↑: increased; ↓: decreased; GPX: glutathione peroxidase; GR: glutathione reductase; H_2_O_2_: hydrogen peroxide; MDA: malondialdehyde; NO: nitric oxide; O_2_: superoxide; ROS: reactive oxygen species; SOD: superoxide dismutase; TAC; total antioxidant capacity; TBARS: thiobarbituric acid reactive substances. Adapted from Powers and Marrocco et al. [31,32] (first two columns) and based on the results of this review (last four columns).

## Appendix A

**Table A1:** Overview of search terms that were used to conduct the literature search.

Chapter	Search Terms ^1^
The Effect of Exercise on Oxidative Stress in Healthy People	“oxidative stress”, “ROS”, “antioxidant capacity”, “exercise”, “physical activity”, “physical training”, “single bout”, “acute”, “intense”, “moderate”
Oxidative Stress Contributes to Pain	“oxidative stress”, “ROS”, “antioxidant capacity”, “chronic pain”, “nociception”, “fibromyalgia”, “chronic fatigue syndrome”, “pain disorders”
Interactions between Oxidative Stress, Exercise and Pain	“oxidative stress”, “ROS”, “antioxidant capacity”, “exercise”, “physical activity”, “chronic pain”, “pain disorders”, “chronic widespread pain”, “exercise-induced hyperalgesia”, exercise-induced hypoalgesia”
The Potential Role of the Autonomic Nervous System in the Interplay between Oxidative Stress, Exercise, and Pain	“oxidative stress”, “ROS”, “autonomic nervous system”, “exercise”, “chronic pain”
Genetic and Epigenetic Regulatory Mechanisms	“oxidative stress”, “ROS”, “epigenetics”, “DNA methylation”, “histone acetylation”, “exercise”, “chronic pain”

^1^ These search terms were used in various combinations. Additional synonyms or related search terms might have been used.
